# Non-neuronal and neuronal BACE1 elevation in association with angiopathic and leptomeningeal β-amyloid deposition in the human brain

**DOI:** 10.1186/s12883-015-0327-z

**Published:** 2015-05-02

**Authors:** Zhi-Qin Xue, Zheng-Wen He, Jian-Jun Yu, Yan Cai, Wen-Ying Qiu, Aihua Pan, Wei-Ping Gai, Huaibin Cai, Xue-Gang Luo, Chao Ma, Xiao-Xin Yan

**Affiliations:** Department of Anatomy and Neurobiology, Central South University School of Basic Medical Science, Changsha, Hunan 410013 China; Department of Anatomy, Xinjiang Medical University, Urumqi, Xinjiang 830011 China; Central South University Affiliated Tumor Hospital, Changsha, Hunan 410006 China; Department of Human Anatomy, Histology and Embryology, Institute of Basic Medical Sciences, Neuroscience Center, Chinese Academy of Medical Sciences, School of Basic Medicine, Peking Union Medical College, Beijing, 100730 China; Department of Surgery and Centre for Neuroscience, Flinders University School of Medicine, Bedford Park, SA 5042 Australia; Laboratory of Neurogenetics, National Institute on Aging, National Institutes of Health, Bethesda, MD 20892 USA

**Keywords:** Amyloidosis, Brain aging, Blood brain barrier, Dementia, Neurodegeneration

## Abstract

**Background:**

Cerebral amyloid angiopathy (CAA) refers to the deposition of β-amyloid (Aβ) peptides in the wall of brain vasculature, commonly involving capillaries and arterioles. Also being considered a part of CAA is the Aβ deposition in leptomeninge. The cellular origin of angiopathic Aβ and the pathogenic course of CAA remain incompletely understood.

**Methods:**

The present study was aimed to explore the pathogenic course of CAA in the human cerebrum via examination of changes in β-secretase-1 (BACE1), the obligatory Aβ producing enzyme, relative to Aβ and other cellular markers, by neuroanatomical and biochemical characterizations with postmortem brain samples and primary cell cultures.

**Results:**

Immunoreactivity (IR) for BACE1 was essentially not visible at vasculature in cases without cerebral amyloidosis (control group, n = 15, age = 86.1 ± 10.3 year). In cases with brain amyloid pathology (n = 15, age = 78.7 ± 12.7 year), increased BACE1 IR was identified locally at capillaries, arterioles and along the pia, localizing to endothelia, perivascular dystrophic neurites and meningeal cells, and often coexisting with vascular iron deposition. Double immunofluorescence with densitometric analysis confirmed a site-specific BACE1 elevation at cerebral arterioles in the development of vascular Aβ deposition. Levels of BACE1 protein, activity and its immediate product (C99) were elevated in leptomeningeal lysates from cases with CAA relative to controls. The expression of BACE1 and other amyloidogenic proteins in the endothelial and meningeal cells was confirmed in primary cultures prepared from human leptomeningeal and arteriolar biopsies.

**Conclusion:**

These results suggest that BACE1 elevation in the endothelia and perivascular neurites may be involved in angiopathic Aβ deposition, while BACE1 elevation in meningeal cells might contribute Aβ to leptomeningeal amyloidosis.

## Background

β-Amyloid (Aβ) peptide deposition in the brain is commonly associated with aging and age-related neurological diseases. Extracellular Aβ deposition in brain parenchyma, especially in the form of neuritic plaques, serves as one of the definitive diagnostic criteria for Alzheimer’s disease (AD) [1,2]. Aβ deposition can also occur in the wall of blood vessels, referred to as cerebral amyloid angiopathy (CAA). Further, β-amyloidosis may present on the pial surface, which is also considered a part of CAA. Overall, CAA coexists frequently with plaque lesions in AD brains [3]. Also of note, postmortem studies show that vascular amyloidosis can occur in the brains of non-demented elderly or patients with vascular dementia and other neurological conditions [4-9]. While CAA may be regarded as a pathological rather than disease entity, its clinical impact is increasingly recognized. CAA is frequently associated with blood brain barrier (BBB) damage and degeneration of vascular components, leading to silent microbleeding or active hemorrhage, which may cause secondary neuronal damage and functional loss [10-14].

The pathogenic mechanism underlying CAA has been conceptualized largely in context of an abnormal Aβ conveyance around the neurovascular interface. Many studies explored the role of reduced brain-to-blood efflux and/or increased periphery-to-central transportation of Aβ in cerebral amyloidosis e.g., [15-18]. Thus, CAA may occur as a consequence of interstitial Aβ elevation in the brain together with impaired drainage of the peptides from parenchyma to blood [19-22]. Retrograde Aβ transportation from blood into the brain is also considered to cause vascular amyloidosis, with some studies explored a role for platelets in Aβ production and the development of CAA [23,24].

β-Secretatse-1 (BACE1) is the obligatory enzyme known for its cleavage to the β-amyloid precursor protein (APP) leading to Aβ production. A role for BACE1 upregulation in parenchymal plaque pathogenesis has been evaluated by multiple groups, including us [25,26]. According to recent cell line [27-29] and postmortem brain studies [30,31], BACE1 elevation in vascular endothelia and smooth muscle cells may play a role in the pathogenesis of CAA. However, evidence for site-specific BACE1 elevation relative to angiopathic Aβ deposition from the human brain is still missing.

We speculated that BACE1 elevation could occur at sites of amyloid angiopathy in the human brain. As such, identifying the cellular elements showing increased BACE1 expression could help reveal the origin of pathologically important Aβ products in the development of CAA. In the present study we choose a cohort of postmortem brains from our recently established human brain banks [32]. BACE1 alteration relative to Aβ and other labeling was determined anatomically and biochemically, with a focus on cerebral vascular and leptomeningeal lesions. For cross-validation, neuritic plaques were examined and documented as a reference system when appropriate. Primary cell cultures were also used to verify the existence of the amyloidogenic machinery in vascular and meningeal cells.

## Methods

### Postmortem human brains and surgical biopsies

Postmortem human brains were obtained through the Willed Body Donation Programs at Peking Union Medical College and Xiangya School of Medicine. Surgical human biopsies were obtained from the Departments of Neurosurgery, Otolaryngology-Head and Neck Surgery of the Affiliated Tumor Hospital of Central South University following informed consent. All experimental procedures were approved by the Institutional Ethics Committee for Research and Education.

Brains were from donors who were not clinically diagnosed with dementia of the AD or vascular type at the time of hospitalization for the care of terminal illnesses. Amyloid and tau pathology were evaluated in sections from the prefrontal and temporal lobe blocks to identify proper samples for the present study. A total of 30 cases were selected and matched into a group with brain amyloid pathology (i.e., parenchymal and vascular amyloid deposition) and a control group that showed no Aβ deposition in the brain, with the amyloid and neurofibrillary lesions scored according to the Braak staging method [33,34] (Table 1). Given a mixed presence of parenchymal and vascular amyloid pathology in individual cases, the former group was referred to as the “amyloidosis” group. Sex, age and postmortem delay were comparable between the two groups. Tau pathology existed in the majority of the “amyloidosis” cases as well as a few control cases (Table 1).

**Table 1:** **Patient information, grouping and Braak scoring**

**Group**	**Case code**	**Postmortem delay (hr)**	**Amyloid plaques**	**Neurofibrillary tangles**
Group with brain amyloid pathology (male: 9, female: 6, age range: 66-95 years)	case 1	5	C	IV
	case 2	5.5	C	V
	case 3	6.5	C	V
	case 4	6	A	N/A
	case 5	8	B	II
	case 6	8	A	N/A
	case 7	5	A	II
	case 8	3.5	B	II
	case 9	4.5	B	III
	case 10	7	B	N/A
	case 11	3	B	V
	case 12	5.5	B	II
	case 13	6	C	IV
	case 14	4.5	B	III
	case 15	5	C	III
Control group (male: 9, female: 6, age range: 58-97 years)	case 1	5	N/A	II
	case 2	5.5	N/A	N/A
	case 3	4.5	N/A	II
	case 4	6.5	N/A	I
	case 5	6	N/A	II
	case 6	4	N/A	N/A
	case 7	7.5	N/A	N/A
	case 8	5	N/A	N/A
	case 9	5	N/A	N/A
	case 10	8	N/A	I
	case 11	8.5	N/A	N/A
	case 12	6.5	N/A	N/A
	case 13	6.5	N/A	N/A
	case 14	4.5	N/A	II
	case 15	8	N/A	N/A
	**P value**	0.285	N/A: not applicable	

### Brain tissue processing

The brains were bisected after removal, with each hemisphere further cut at the frontal plane into approximately 2 cm-thick slices. The slices from one half-brain were fixed by immersion in 4% paraformaldehyde in 0.1 M phosphate-buffered saline (PBS) for 4 days at 4°C, with the slices of the other half-brain stored at -70°C. A prefrontal (~6 cm from the frontal pole) and two temporal slices (passing the amygdala and mid-hippocampus) were taken from the fixed half-brains, and then placed in 10-30% sucrose in 0.01 M phosphate buffer for cryoprotection. These brain blocks were cut at the frontal plane into sections at 40 μm-thickness in a cryostat. For each block, twelve sets of sections were collected into 6-well culture plates, with each set containing 10 sections with equal intervals (~500 μm apart). Sections were washed with PBS for 3 times to remove the embedding medium before they were transferred into a cryoprotectant and stored at -20°C until histological studies.

### Preparation of postmortem leptomeningeal samples

Leptomeninge over the temporal lobes was peeled off with a pair of fine forceps after brain removal, and rinsed thoroughly in cold PBS. Meningeal blood vessels identifiable under the dissection microscope were dissected out and collected separately. For each case, the leptomeningeal vascular and the relatively “pure” meningeal samples were divided into two parts, with the first fixed in 4% paraformaldehyde overnight then processed histologically as described above for the brain samples, except that the sections were cut at 20 μm in thickness and thaw-mounted on microslides. The remaining samples were stored frozen (-70°C) until being used for biochemical assays.

### Preparation of peripheral human vascular biopsies

Small arteries and veins ranging 0.5-2 mm in diameter were obtained at surgery from patients (n = 8, males, 37-60 year-old) suffering from benign thyroid lesions. Vessels were isolated from subcutaneous or intermuscular fascia before exposure and resection of the thyroid tissue. A small part of peripheral vascular biopsy was fixed in 4% formaldehyde, and used for histological examination. The remaining part was collected in ice-old Dulbecco’s modified Eagle’s medium with Nutrient Mixture F12 (DMEM/F12; Life Technologies Corporation, Shanghai, China), and used for biochemical (n = 4) and primary cell culture (n = 4) studies.

### Preparation of leptomeningeal biopsies

Leptomeningeal biopsies were obtained at surgery from six patients suffering from deep brain glioma (males; 47-63 year-old). The leptomeninge over the cortex to be resected was peeled off under surgical microscope, before the underlying cortical tissue was aspirated. The leptomeningeal samples were collected in ice-old Dulbecco’s modified Eagle’s medium with Nutrient Mixture F12 (DMEM/F12; Life Technologies Corporation, Shanghai, China), and used primarily for primary cell culture, with a few arterioles dissected out in each case for anatomical study as well.

### Primary vascular and meningeal cell culture

Resected small arteries and leptomeninge samples were digested in 0.25% Trypsin-EDTA solution (Catalog #25200-072; Life Technologies Corporation) and centrifuged at 15,000 g for 10 minutes. The resulting cell pellets were re-suspended with DMEM/F12 containing 10% fetal bovine serum, adjusted to a beginning cell density approximately 5 × 10^6^ cells/mL. Cells were cultured at 37°C in a humidified atmosphere containing 95% air and 5% CO2, in Corning-Costar plates (Catalog #CLS3516; Life Technologies Corporation). Each well was loaded with 2 ml of medium and a glass coverslip coated with polylysine, with the medium changed every 2 days.

### Primary antibodies

A set of well-characterized primary antibodies were used in the present study for immunohistochemistry and immunoblot (Table 2). These included antibodies for profiling AD-type neuropathology (Aβ, APP, BACE1, phosphorylated-tau), determination of neuronal (MAP2, β-tubulin, synaptophysin), vascular (collagen-IV), endothelial (CD31) and smooth muscle components (smooth muscle actin, αSMA), as well as reference proteins (β-actin and GAPDH).

**Table 2:** **Primary antibodies used in the present study**

**Antibody**	**Source**	**Product #**	**Dilution**
Mouse anti-Aβ1-42, 12 F4	Signet	39240	(1:2000)
Mouse anti-Aβ1-16, 6E10	Signet	39320	(1:4000)
Rabbit anti-β-actin	Sigma-Aldrich	A2066	(1:5000)
Mouse anti-amyloid precursor protein (APP), 22C11	Millipore	MAB348	(1:1000)
Rabbit anti-amyloid precursor protein (APP), C-terminal	Edward Koo	CT15	(1:2000)
Rabbit anti-amyloid precursor protein (APP), C-terminal	Serotec	AHP538	(1:2000)
Mouse anti-alpha-smooth muscle actin (αSMA)	abcam	ab7817	(1:2000)
Rabbit anti-BACE1α (a.a. residues 46-163)	Huaibin Cai	anti-BACE1α	(1:2000)
Mouse anti-CD31	Dako	M0823	(1:1000)
Mouse anti-collagen, type IV	Sigma-Aldrich	C1926	(1:1000)
Mouse anti-fibronectin	Sigma-Aldrich	F7387	(1:2000)
Mouse anti-glyceraldehyde 3-phosphate dehydrogenase (GAPDH)	Millipore	MAB374	(1:10000)
Mouse anti-glial fibrillary acidic protein (GFAP)	Millipore	MAB3402	(1:2000)
Mouse anti-microtubule associated protein-2 (MAP2)	Sigma-Aldrich	M9942	(1:1000)
Rabbit anti-presenilin-1 N-terminal fragment (PS1-NTF)	Samuel Gandy	Ab14	(1:500)
Rabbit anti-phospho-Tau (*p*-Ser199/Ser202) (p-Tau)	Sigma-Aldrich	T6819	(1:3000)
Mouse anti-synaptophysin (SYN)	Millipore	MAB329	(1:4000)
Rabbit anti-β-tubulin-III	Sigma-Aldrich	T2200	(1:10000)

### Immunohistochemistry and immunocytochemistry

Sections from multiple cases were stained histologically and immunohistochemically under identical conditions in each experiment. Antigen retrieval was applied for BACE1 labeling, with 50% formamide and 50% 2 × SSC (0.3 M sodium chloride and 0.03 M sodium citrate) at 65°C for 1 hour; and for Aβ antibody labeling, with 50% formic acid in PBS for 30 minutes at room temperature. Immunostaining with the ABC method began with a treatment of sections in 5% H_2_O_2_ in PBS for 30 minutes, followed by a pre-incubation in 5% normal horse serum in PBS with 0.3% Triton X-100 for 1 hour. Incubations with the primary antibodies were carried out at 4°C overnight with gentle agitation, after which sections were reacted with a biotinylated pan-specific secondary antibody (horse anti-mouse, rabbit and goat IgGs) at 1:400 for 2 hours. Following reaction with the ABC reagents (1:400) (Vector Laboratories, Burlingame, CA, USA) for 1 hour, immunoreactivity was developed in 0.003% H_2_O_2_ and 0.05% 3,3’-diaminobenzidine (DAB). Brain sections were washed 3 times, 10-minute each, between antibody incubations.

Double immunofluorescence was initiated with an incubation of the sections in PBS containing 5% donkey serum for 30 minutes, followed by reaction with a pair of primary antibodies raised in different species (Table 2) overnight at 4°C. On the second day, sections were incubated at room temperature for 2 hours with Alexa Fluor® 488 and Alexa Fluor® 594 conjugated donkey anti-mouse, anti-rabbit or anti-goat IgGs (1:200, Invitrogen, Carlsbad, CA, USA). Sections were further treated with the autofluorescence eliminator (Catalog #2160, Millipore, Billerica, USA), counter-stained with bisbenzimide (Hoechst 33342, 1:50000, Catalog #B2261, Sigma-Aldrich) and mounted with anti-fading medium. For immunocytochemistry, cells grown on glass coverslips were fixed with 4% paraformaldehyde for 30 minutes. Single or double immunofluorescence was carried out on-slide to determine the expression of amyloidogenic and specific vascular cell proteins using the protocols described above for brain tissue staining.

### Histology and histochemistry

Some immunolabeled sections were counterstained with toluidine blue or cresyl violet to facilitate histological orientation or graphic illustration. A few BACE1/Aβ antibody immunostained sections from each case were further processed with Perl’s Prussian blue stain to visualize ferric iron leakage/deposition. In addition, at least two temporal lobe sections from each brain were initially processed for nicotinamide adenine dinucleotide phosphate diaphorase (NADPH-d) histochemistry. Sections showing optimal neuronal NADPH-d reactivity were dual-stained for BACE1 and Aβ [35,36].

### Western blot

Postmortem leptomeningeal samples, vascular biopsies and cultured cells were homogenized by sonication in T-PER extraction buffer (Pierce, Rockford, IL, USA) containing protease inhibitors (Roche, Indianapolis, IN, USA). The lysates were centrifuged at 15,000 ***g***, with supernatants collected and protein concentrations determined by DC protein assay (Bio-Rad Laboratories, Hercules, CA, USA). Extracts containing 50 μg protein were run in 8%, 14% or 20% SDS-polyacrylamide gel electrophoresis (PAGE) gels. Separated proteins were electrotransferred to Trans-Blot pure nitrocellulose membranes (Bio-Rad Lab.). The nitrocellulose membrane strips containing the target proteins were immunoblotted with antibodies to APP (22C11), APP β-CTF or C99 (AHP538, 6E10), BACE1, PS1-NTF (ab14), β-tubulin-III, β-actin or glyceraldehyde-3-phosphate dehydrogenase (GAPDH). The membranes were further reacted with HRP-conjugated goat anti-rabbit or anti-mouse IgGs (1:20000; Bio-Rad Laboratories), with protein signal visualized with the ECL-Plus Western Blotting Detection Kit.

### APP β-site cleavage (BACE1) activity assay

Activities of β-site APP cleavage in tissue/cell lysates were measured in duplicate in 96-well transparent flat-bottomed plates. Samples were homogenized on ice as described above for western blot analysis, and assayed for enzyme activity using a commercial kit (Calbiochem, La Jolla, CA, USA, Catalog #565785), following the manufacturer’s instructions. The fluorescent signal was captured in a Bio-Rad microplate reader (PR 3100 TSC).

### Enzyme linked immunosorbent assay for Aβ

Aβ42 levels (pM/ml) in leptomeningeal lysates were assayed in duplicate by solid sandwich enzyme-linked immunosorbent assay (ELISA) using a commercial kit (Novex®KHB3442 for Aβ42) following the manufacturer’s instruction (Life Technologies Corporation). Signals were captured in the Bio-Rad microplate reader (PR 3100 TSC). Aβ concentrations were calculated according to the standard curve generated from the readouts of synthetic peptides.

### Imaging, data analysis and figure preparation

Sections were examined on an Olympus fluorescent BX53 microscope equipped with a digital imaging system (CellSens Standard, Olympus Corporation, Japan), and on a confocal microscope (Nikon-EZ-C1, Japan). Confocal images were processed with the Nikon-EZ-C1 Viewer software. Images were taken using 2× to 40× objectives, with low magnification images montaged for presentation. All confocal immunofluorescence images were taken with the 20 × objective by three scanning of tissue with each in a depth of 1.9 μm. Images prepared for densitometric analysis were obtained at once following a single batch of immunohistochemical preparation, with the same exposure setting applied for all photo documentation. A correlated densitometry was designed for comparing BACE1, 6E10 and bisbenzimide labeling at individual intracortical vascular profiles in (the methodology will be detailed later with illustration). Optic densities of protein bands were obtained on immunoblot images, with the data normalized to the internal references.

Densitometric and numerical data were processed for group comparison by calculation of means and standard derivations (SD), with normalization to appropriate reference applied. Statistical comparison of means were performed using Student-*t* test, or one-way ANOVA with posthoc test (Prism GraphPad, San Diego, CA, USA), with the minimal significant level of difference set at p < 0.05. Figures were assembled with Photoshop 7.0.

## Results

### Identification and morphological characterization of vascular BACE1 labeling

**Figure 1:**
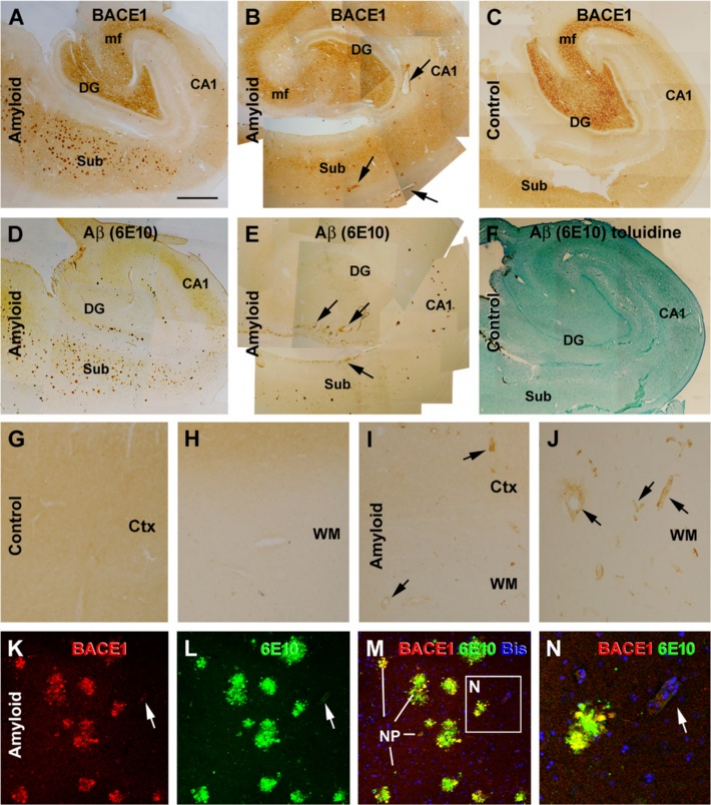
Representative images showing increased β-secretase (BACE1) immunoreactivity (IR) at selected vascular sites, in addition to neuritic plaques, in the human brains with amyloid deposition relative to controls. Panels **(A-F)** are montages of low magnification images showing BACE1 **(A-C)** and 6E10 [reactive to Aβ, potentially to β-amyloid precursor protein (APP) and APP β-C-terminal as well] **(D-F)** IR over the subiculum (Sub), hippocampus (CA sectors) and dentate gyrus (DG) between adjacent sections from two cases with brain amyloid pathology **(A,B,D,E)** and one control **(C,F)**. In the second case **(B,E)**, vascular BACE1 and 6E10 labeling (arrows) is evident at low magnification. In the control case, no staining is seen with 6E10 labeling (F, with tissue lamination illustrated by toluidine counterstain). Note that the BACE1 IR at the mossy fiber terminal field is comparable in **(A-C)**. Panels **(G-J)** show low magnification views of BACE1 IR in the temporal neocortical grey and white matter from two additional brains, with a diffuse neuropil pattern in the control **(G,H)** and increased labeling in the amyloid case at selected vascular profiles (I.J). Confocal double immunofluorescence shows a partial colocalization of BACE1/6E10 IR among typical neuritic plaques **(K-N)**, with the structures exhibiting overlapped labeling (appearing yellow) representing dystrophic neurites **(M,N)**. A capillary profile exhibiting weak BACE1/6E10 IR is also seen in the field **(M, N)**. Scale bar in **(A)** = 2.5 mm, applying to **(B-F)**, equivalent to 500 μm for **(G-J)**, 200 μm for **(K-M)** and 50 μm for **(N)**.

Increased BACE1 immunoreactivity (IR) relative to parenchymal amyloid pathology has been reported in animal and human brains [32,35,36]. Consisted with the existing data, increased BACE1 and Aβ IR were found in the cases with brain amyloid pathology as compared to controls (Figure 1). Briefly, in the cases of the amyloidosis group, BACE1 IR was increased at local sites against an overall neuropil-like background (Figure 1A,B,D,E). In contrast, BACE1 IR in control cases exhibited essentially a diffuse neuropil-like pattern across the temporal lobe cortical structures (Figure 1C,F). The distinct BACE1 labeling normally present at the mossy fiber terminal field was otherwise comparable between cases with and without cerebral amyloid pathology (Figure 1A-C).

**Figure 2:**
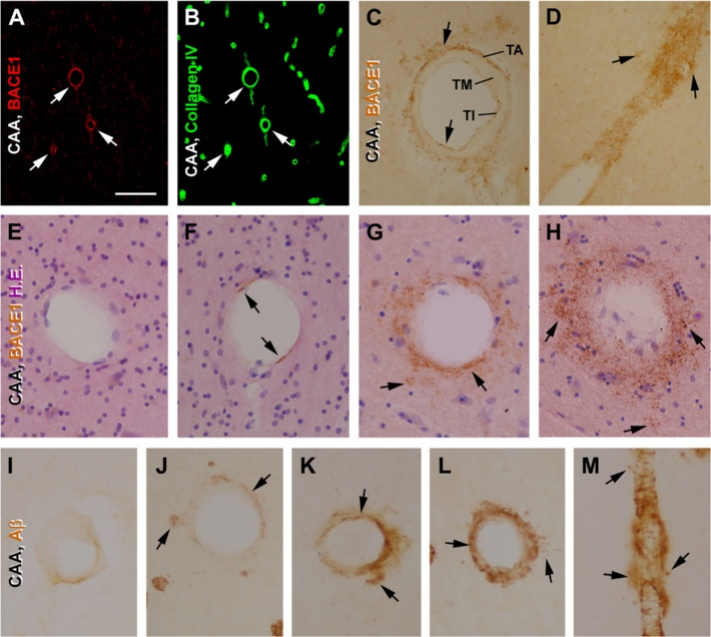
Morphological characterization of vascular (arteriolar) BACE1 and 6E10 immunoreactivity (IR) in postmortem human temporal neocortex. Panels **(A,B)** show selective BACE1 labeling at cortical vasculature visualized by collagen IV. Panels **(C)** illustrates BACE1 IR at a cross-sectioned arteriole, localizing to the tunica interna (TI) and around the tunica adventitia (TA) or the perivascular area, but not in the tunica media (TM). Panel **(D)** shows a feather-like pattern of BACE1 IR along a longitudinally cut intracortical arteriole. Panels **(E-H)** illustrate variable patterns of BACE1 IR among labeled **(F-H)** relative to unlabeled **(E)** arterioles in immunohistochemical preparation with hematoxylin and eosin stain (H.E.) counterstain. Arrows points to BACE1 IR in the TI **(C,F)**, TA **(C,D)** and TM **(G,H)**. Loss of cells in the vascular wall is noticeable in **(G)** and **(H)** relative to **(E)** and **(F)**. The BACE1 IR in the TA and TM appears process-like **(C,D,G,H)**. Panels **(I-L)** illustrate variable patterns of Aβ labeling at arterioles, with Aβ IR occurs primarily at the TI **(I)** or in the TM **(J-L)**. Perivascular Aβ IR (arrows) often co-exists among the profiles with Aβ deposition in the TM with varying intensity **(J-L)**, and appears segmentally in longitudinal view of the labeled vessel **(M)**. Scale bar = 500 μm in **(A)** applying for **(B)**; equal to 200 μm for **(M)** and 100 μm for other panels.

Importantly, while essentially no vascular BACE1 IR was identifiable in sections from the control group (Figure 1C,G,H), increased BACE1 IR was seen at some vascular profiles in the cortex and hippocampal formation in the cases with neuritic amyloid pathology (Figure 1B,E,I,J). In general, the increased vascular BACE1 IR could occur around the pia (will be detailed in following section), in any cortical layer or in the white matter. The labeled vessels varied in size, but appeared to be mostly capillaries and arterioles as judged on the basis of their size, histological configuration and laminar distribution. This site-specific increase of BACE1 IR at capillary and arteriole-like profiles was confirmed by double immunofluorescence for BACE1/6E10 (Figure 1K-N) and BACE1/collagen IV (Figure 2A, B). At higher magnifications, BACE1 IR at blood vessels exhibited variable patterns and intensities in DAB (Figure 2C,D) or DAB/H.E. dual-stain (Figure 2E-H) preparations, better appreciated in the latter wherein the vascular lamination was displayed. Specially, BACE1 IR at arteriole-like profiles showed a differential laminar distribution that was related to the overall amount of labeling at individual vessels. Thus, when comparing intracortical arterioles without (Figure 2E) to those with BACE1 IR (as assessed in the same sections), the labeling appeared to occur initially then intensify in the tunica intima (TI) or endothelial layer (Figure 2F). As the endothelial BACE1 IR became evident, labeled elements often occurred in the perivascular area, with the tunica media (TM) or the smooth muscle layer spared (Figure 2C). As the overall BACE1 IR became more abundant, the labeling occurred also in the TM (Figure 2G,H). BACE1 IR in and surrounding the vascular wall might appear segmentally along the vessels cut longitudinally, giving the profile a feather-like appearance (Figure 2D). It should be noted that BACE1 labeled elements in the (TM) appeared as fine processes, some had local swellings (Figure 2G,H), suggestive of a labeling to neuronal terminals than smooth muscle cells.

The vascular Aβ IR exhibited a differential labeling pattern in ABC-DAB immunohistochemical preparations. As defined by some neuropathologists [13], vascular Aβ deposition could be staged according to the pattern and amount of Aβ IR between individual vessels, which was applicable to the intracerebral arterioles exhibiting 6E10 IR in this study (Figure 2I-M). Thus, light 6E10 IR appeared to initially occur at the TI or endothelial layer (Figure 2I), followed by the emergence and intensification of the labeling in the TM (Figure 2J-L). However, we observed perivascular 6E10 IR at arterioles with fairly light or heavy Aβ IR in the vascular wall (Figure 2J-K). The profiles containing vascular and perivascular Aβ IR resembled the so-called “dysphoric or dyshoric CAA” defined in previous studies [13,37,38].

### Correlative analysis of vascular BACE1 and Aβ antibody labeling

**Figure 3:**
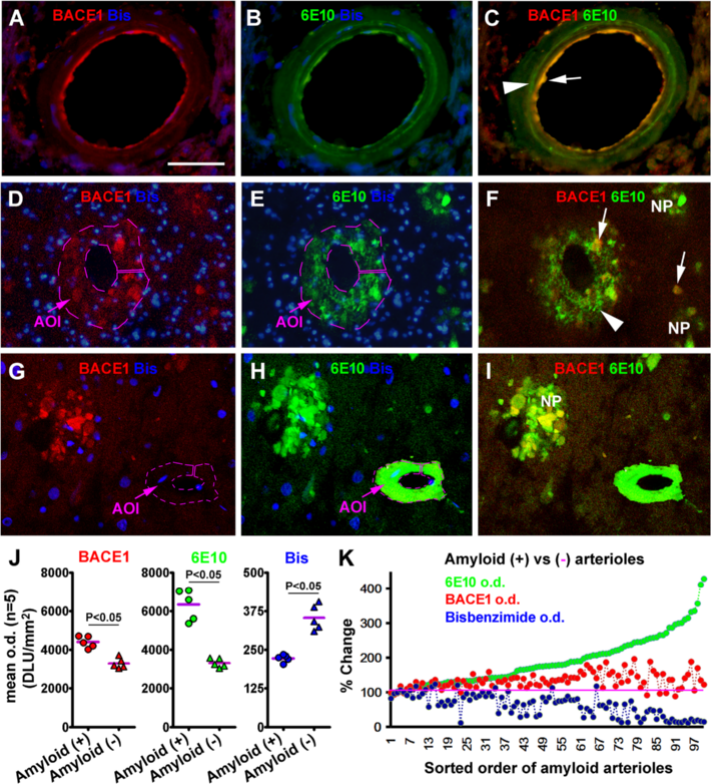
Correlative morphometric characterization of BACE1 and 6E10 immunoreactivity (IR) in intracortical arterioles in confocal fluorescent preparation. Panels **(A-C)** show colocalized BACE1/6E10 IR in the endothelia (arrows), with a ring-like Aβ deposition (arrowhead) in the smooth muscle layer that is not colocalized with BACE1 IR. Panels **(D-F)** demonstrate an arteriole containing BACE1 labeled neurites and local Aβ deposition (arrowheads) in the vascular wall, with BACE1 IR colocalizing with 6E10 IR inside but not outside swollen process-like elements (arrows). Panels **(H-J)** show a vessel with densely packed Aβ products in the wall, with BACE1 IR over the same area comparable to background. Fusiform bisbenzimide (Bis)-labeled nuclei appear lost in the middle layer of arterioles in **(D/E, G/H)** relative to **(A/B)**. A typical neuritic plaque (NP) consisted of BACE1 labeled dystrophic neurites and Aβ deposits is also seen in **(G-I)**. Panels **(J)** plots the mean optic densities (o.d.) of BACE1, 6E10 and bisbenzimide fluorescence measured over the wall of individual normal [i.e., amyloid negative (-), 10/brain)] and amyloid [i.e., amyloid positive (+), 20/brain] arterioles from five individual brains, with the area of interest (AOI) illustrated as the purple-line circled areas in (**D/E** and **G/H**, and also in Figure 4A to demonstrate the method to measure densities at normal control vessels). Note the increase of BACE1/6E10 o.d. and reduction of bisbenzimide o.d. in the amyloid (+) relative to (-) arterioles. Panel **(K)** shows a correlative densitometric analysis by sorting the order of individual vessels (n = 100 from 5 brains) according to their elevated levels of 6E10 densities relative to baseline (defined as 100%, mean of densities reported from all non-amyloid vessels). BACE1 density increases initially in parallel with that of 6E10 IR, but tends to reduce among most profiles as 6E10 IR further increases. Bisbenzimide density tends to decline with the increase of 6E10 labeling. Scale bar = 50 μm in **(A)** applying to **(B,** **C,G-I)**, equivalent to 100 μm for **(D-F)**.

In double immunofluorescence, a distinct endothelial BACE1/6E10 colabeling was identified at intracerebral arterioles, with some exhibited 6E10 IR in the TM arranged as a ring-like pattern in the absence of BACE1 IR (Figure 3A-C). Among the intracortical arterioles displaying BACE1/6E10 IR in the middle wall, a pattern of partial colocalization was recognized (Figure 3D-F). At some intracortical vessels with heavy 6E10 IR, the intensity of BACE1 IR was otherwise at the background levels (Figure 3G-I). As a reference, typical neuritic plaques showed a partial colocalization pattern as well, with BACE1/6E10 IR overlapped only in the dystrophic neurites that were surrounded by 6E10 reactive Aβ deposits (Figure 3G-I). The differential colocalization of BACE1 relative to intracellular vs. extracellular 6E10 IR might be related to a cross-reactivity of 6E10 to intraneuronal APP and/or APP β-C terminal fragments, although the 6E10 IR inside the dystrophic neurites could reflect the existence of intracellular Aβ [25].

**Figure 4:**
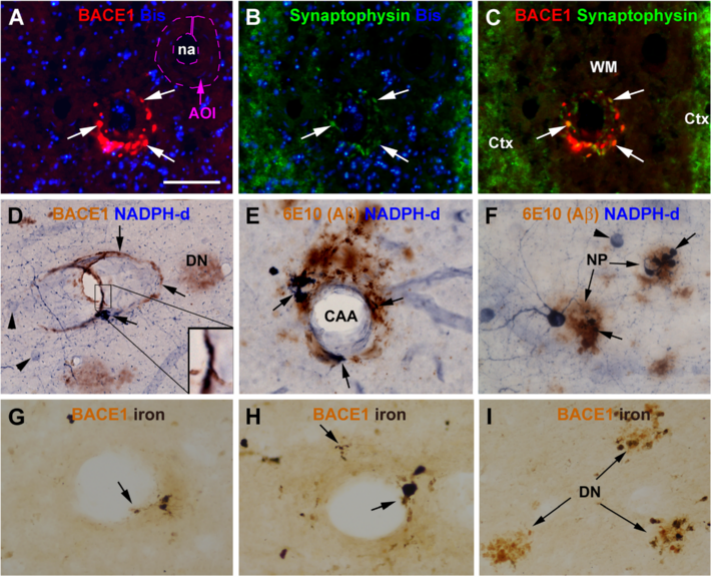
Neuritic profiles inside and surrounding the wall of intracerebral arterioles from cases with brain amyloid pathology. Panels **(A-C)** show a partial colocalization of BACE1 and synaptophysin immunofluorescence in the vascular wall of an arteriole in the subcortical white matter (WM) (arrows pointing to colocalized parts), with a nearby arteriole (circled with broken purple line) exhibited background signal for both markers. Panels **(D)** shows BACE1 labeled dystrophic neurites (arrows) partially colabeled (enlarged insert) for nicotinamide adenine dinucleotide phosphate diaphorase (NADPH-d) surrounding an intracortical vessel, with clusters of dystrophic neurites (DN) in the field. Panel **(E)** shows NADPH-d positive dystrophic neurites (arrows) in and around the wall of a vessel with Aβ deposits. Panel **(F)** shows rosette-like NADPH-d positive dystrophic neurites in compact amyloid plaques (NP). Panels **(G,H)** show BACE1 labeled neurites in small **(G)** and larger **(H)** amounts in the vascular wall that are partially colabeled by Prussian blue, as are those organized as rosset-like clusters (I). The colabeled parts appear grey to black because of the brown DAB background color. The AOI label in **(A)** illustrates a method to measure optic density in normal arterioles (referring to Figure 3K-L). Scale bar = 100 μm in **(A)** applying to **(B-I)**.

In ABC-DAB immunohistochemical preparations, cell loss was readily noticeable in the wall of the intracortical arterioles with relatively strong BACE1 IR (compare Figure 2G,H to E,F). We also noticed a loss of bisbenzimide labeled nuclei in the vascular wall of vessels with moderate to strong BACE1/6E10 IR in double immunofluorescence. In order to assess whether there existed a trend of relative changes in BACE1 IR, 6E10 IR and cell loss, we carried out correlative densitometry for the 3 variables among individual cortical and white matter arterioles. This was conducted in batch-processed immunofluorescent sections from 5 cases. While scanning over the cortex and white matter (following an alternating path), each 6E10 labeled arterioles encountered was imaged. A set of images [the red (BACE1), green (6E10) and blue (bisbenzimide) channel] from the same brain was then put together, converted into a black and white tiff file. Using the OptiQuant software, a measuring template was created in the original green image over the area occupied by 6E10 IR at the blood vessel. This same template was copied and pasted over the other two images and aligning to the same vessel (see Figure 3D/E and G/H for methodological illustration). The optic densities for the 3 types of labeling at the same vascular profile were thus obtained. For a given brain, 20 cross-sectioned intracerebral arteriolar profiles were analyzed by this method. To obtain the baseline densities, 10 cross-sectioned intracortical arterioles not labeled by BACE1 and 6E10 encountered in the same microscopic fields were also photographically documented in each brain. The “immunofluorescent” (actually the background) and bisbenzimide densities were obtained by the same method, except that the vascular measuring template was created in the bisbenzimide image (for a methodological demonstration see the framed artery in Figure 4A).

Among a total of 100 quantified amyloid arterioles, the mean optic densities were 4397.9 ± 298.4 DLU/mm^2^ for BACE1 IR, 6328.6 ± 794.9 DLU/mm^2^ for 6E10 IR and 222.5 ± 11.9 DLU/mm^2^ for bisbenzimide fluorescence. For the 50 control arterioles (10 profiles/brain from the same 5 brains), the mean densities were 3298.3 ± 268.5, 3317.1 ± 237.0 and 253.4 ± 41.3 DLU/mm^2^ for BACE1, 6E10 and bisbenzimide signals, respectively (Figure 3J). There was a significant difference between the amyloid and control arterioles for the mean density of BACE1 IR (P < 0.0001), 6E10 IR (P = 0.0003) and bisbenzimide (P = 0.0001) (two-tailed Student-*t* test). To explore a trend of change between the 3 variables, the density of each marker measured at a given vessel was normalized to the mean density of the corresponding marker from all control profiles (i.e., defined as 100% or baseline). The order of the vascular profiles was then resorted according to the normalized 6E10 densities (from low to high), with the 3 variables plotted as a function of the vascular order numbers (Figure 3K). BACE1 and 6E10 IR concurrently increased initially, with BACE1 IR further increased to approximately at a plateau as the levels of 6E10 IR continued to rise until about 2 fold of the baseline. While 6E10 IR increased further up to 4 fold of the baseline density, BACE1 IR did not continue to elevate but tended to drop towards the baseline among many profiles. Bisbenzimide density tended to decrease with the increase in 6E10 IR, especially among the vascular profiles with 6E10 density reached and above 2 fold of the baseline level (Figure 3K).

### Cross-validation of abnormal neurites inside and apposing to vascular wall

The aforementioned BACE1 immunoreactive elements in the perivascular area and TM were of particular interest as they could imply other cellular contributors than the endothelial cells (verifiable simply by regular histology) of Aβ to amyloidosis. Using double immunofluorescence, the process-like BACE1 immunoreactive elements were found to partially colocalize with synaptophysin (Figure 4A-C). These elements were not labeled with αSMA, a marker of vascular smooth muscle cells (data not shown). The incomplete BACE1/synaptophysin colocalization was considered to indicate that BACE1 elevation was not limited to (dystrophic) presynaptic terminals. As a cross-validation for this latter possibility, we used another neuronal marker, NADPH-d, which could display neuronal processes extensively [39-41]. Following an initial histochemical screening, cases with optimal neuronal NADPH-d reactivity were subjected to further BACE1 and 6E10 immunolabeling. NADPH-d reactive dystrophic neurites were observed in the vascular wall or the perivascular zone, coexisting with BACE1 (Figure 4D) or in mix with local Aβ deposition (Figure 4E). NADPH-d reactive dystrophic neurites were also present in some amyloid plaques (Figure 4F).

Considering the well-established link between CAA and microbleeding [10-12], we explored whether vascular profiles with BACE1 labeled neurites might be associated with hemosiderin leakage. In sections counterstained with Prussian blue, ferric iron deposition was seen in the wall as well as at perivascular area among the arterioles with BACE1 labeled neuritic profiles (Figure 4G-I). There were BACE1 immunoreactive neurites directly colabeled by Prussian blue, with some extended to near the vascular cavity, even in arterioles with a small amount of dystrophic neurites (Figure 4G,H). Iron deposition was also seen at subset of BACE1 labeled dystrophic neurites arranged as rosette-like clusters (Figure 4I), which would represent the sites of parenchymal neuritic plagues.

### Characterization of amyloidogenic protein expression in vascular biopsy and cultured cells

As with previous animal and human brain studies [35,36,42,43], BACE1 IR was essentially not detectable at cerebral vasculature in brain sections from the control cases or at normal vessels in brain sections with amyloid pathology. This would appear not surprising in general given that the differentiation of specific immunolabeling depends on a sufficient signal to background ratio. In other words, since BACE1 is more abundantly expressed in neuronal than vascular elements, neuronal BACE1 IR could mask vascular BACE1 IR, if any, in brain sections under normal conditions. Because “de novo” BACE1 IR was identified at selected vascular sites in postmortem human brain in the present study, we speculated that vascular cells should express BACE1 and perhaps other Aβ producing proteins endogenously.

**Figure 5:**
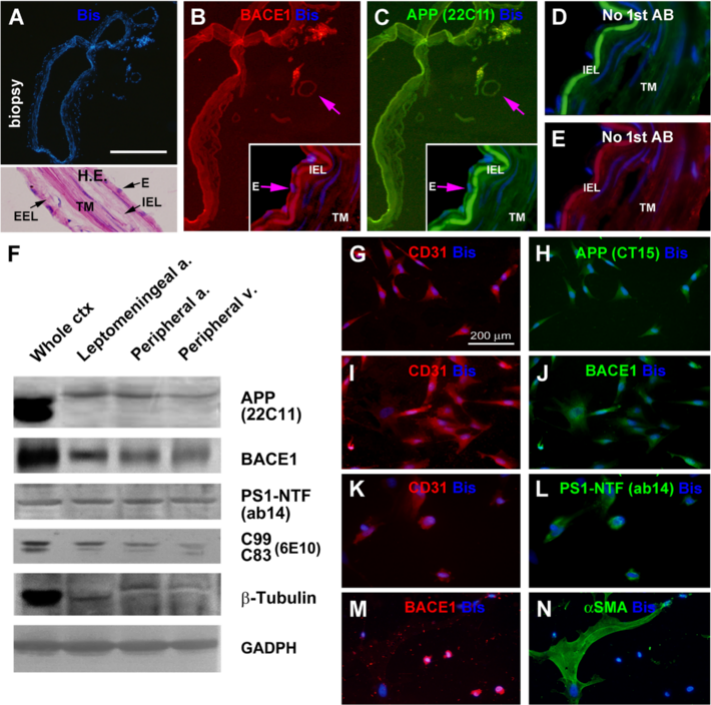
Characterization of the expression of amyloidogenic proteins in vascular biopsy and primary vascular cell culture. Panels **(A-E)** show the expression of the β-amyloid precursor protein (APP) and BACE1 in leptomeningeal biopsy containing a middle-sized and several small arteries (arrow), and capillaries. The endothelial cells (E→) expressed specific APP and BACE1 immunoreactivity **(B,C)**, which can be eliminated by excluding the primary antibodies (1^st^ AB) in immunofluorescent processing **(D,E)**. Autofluorescence exists in the inner elastic lamina (IEL), tunica media (TM) and external elastic lamina (EEL) (refer to the H.E. stained inset in A). Panel **(F)** shows immunoblot characterization of the amyloidogenic proteins in extracts of isolated leptomeningeal arteries, peripheral arteries and veins, with cortical extract (from a control case) used as assay control (50 μg protein loading in each lane). APP, BACE1, presenilin-1 N-terminal fragments (PS1-NTF) and APP β-cleavage products are present in the vascular homogenates. Note that the vascular APP migrates at a higher molecular weight position relative to the brain counterpart. The vascular samples contain minimal amount of β-tubulin relative to cortical lysate. Panels **(G-L)** illustrate immunocytochemical labeling of APP, BACE1 and PS1-NTF in cultured vascular cells expressing CD31, a marker for vascular endothelia. A large cellular profile (appeared as fused cells) is labeled with the smooth muscle cell marker, α-smooth muscle actin (αSMA), but exhibits little BACE1 immunoreactivity **(M,N)**. Scale bar = 500 μm in **(A)** applying to **(B,C)**; equal to 250 μm for **(H-I)** and 50 μm for **(E-G)**.

Surgically isolated blood vessels and primary cell culture, together with human cortical extracts as control, were used to explore the expression of amyloidogenic proteins in adult human vascular cells. In biopsied leptomeningeal and peripheral (image not shown) arteriolar, venular (image not shown) and capillary preparations, BACE1, APP and PS1 (not shown) IR were detectable by immunohistochemistry, localized largely to the endothelial cells (Figure 5A-C). It should be noted that, in relatively large arteries [i.e. with an internal elastic lamina (IEL)], strong and weak fluorescent signal existed in the IEL and TM, respectively (Figure 5B,C). By excluding the primary (Figure 5D,E) or secondary (image not shown) antibodies, the endothelial BACE1 and APP IR became eliminated, while the fluorescent signal in the IEL and TM largely remained, as compared with that in the normally processed sections (Figure 5B,C, inserts). Thus, the fluorescent signal in the endothelial layer appeared to be specific, whereas that in IEL and TM was largely autofluorescence.

Protein products of APP, BACE1 and APP C-terminal fragments were detected in the extracts of the leptomeningeal and peripheral vascular biopsies, at lower levels relative to the cortex (Figure 5F). Notably, vascular APP holo-proteins migrated at a slightly heavier molecular weight position relative to brain APP. Presenilin-1 N-terminal fragments (PS1-NTF), an active component of γ-secretase complex, were also detected in vascular lysates, although at similar levels between the cortical, arterial and venous samples. The vascular samples showed an expected minimal amount of the β-tubulin proteins as compared to cortical lysates (Figure 5F). Vascular extracts were also found to exhibit APP β-site cleavage enzymatic activity, and contain detectable amounts of Aβ42 (data not shown). Immunocytochemical characterization on the expression of amyloidogenic proteins was carried out in primary cell cultures of resected peripheral arteriolar samples maintained in vitro for 4-8 days. Cells expressing CD31, a marker of the endothelia, were found to co-express APP, BACE1 and PS1-NTF IR (Figure 5G-L). Cells expressing the smooth muscle actin ∀SMA, including some appearing in a fused configuration, exhibited minimal BACE1 IR (Figure 5M,N).

### Characterization of BACE1 elevation in association with leptomeningeal amyloidosis

**Figure 6:**
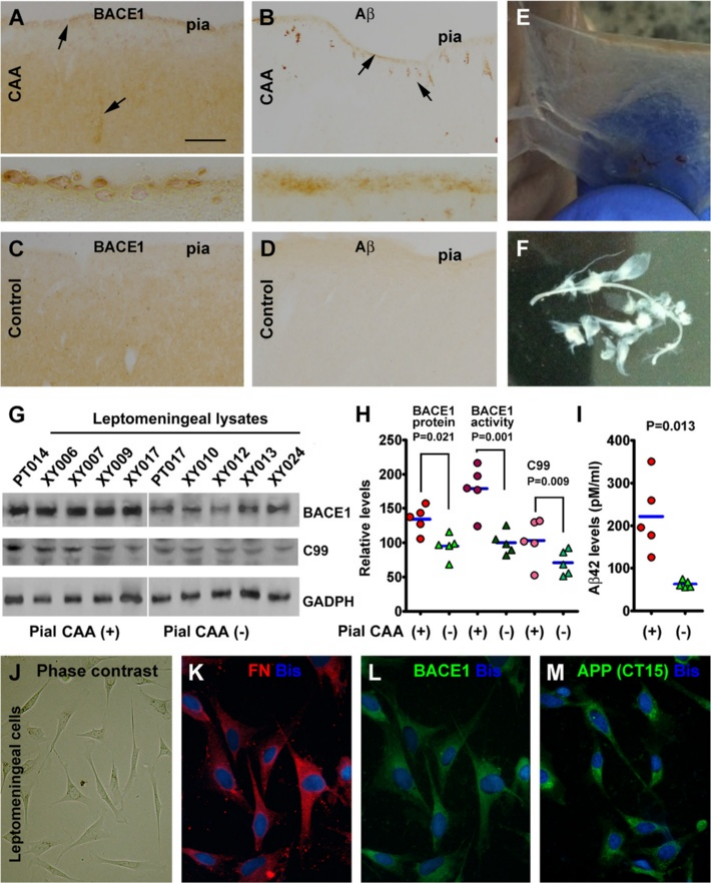
Characterization of BACE1 elevation in meningeal cells associated with leptomeningeal amyloidosis. Panel **(A)** shows BACE1 labeled cells aligning along the pia mater in a case with cerebral amyloid angiopathy with prominent leptomeningeal amyloidosis **(B)**, in contrast to a control case wherein no BACE1 and Aβ (6E10) labeling is present at the pia **(C,D)**. Arrows in **(A,B)** points to profiles with increased BACE1 and 6E10 IR over the background. Panel **(E)** shows a preparation of leptomeninge sample from the temporal lobe **(E)**, which are rinsed thoroughly in cold phosphate buffer for biochemical analysis **(F)**. Levels of BACE1 protein and C99 in leptomeningeal lysates are elevated in 5 cases with CAA relative to 5 control cases **(G,H)**, as is BACE1 enzymatic activity measured in the lysates **(H)**. Aβ42 levels are also higher in the CAA group relative to control **(H)**. In primary cell culture of leptomeningeal biopsies, meningeal cells appear polygonal under phase contrast microscope **(J)**, co-express fibronectin (FN) **(K)** and BACE1 **(L)**. These cells also express the β-amyloid precursor protein (APP) (H, the corresponding FN labeling image is not shown). Scale bar = 250 μm in **(A)** applying to **(B-D)**, equivalent to 50 μm for inserts in **(A,B)**, 25 μm for **(J)** and 12.5 μm for **(K-M)**.

In addition to the above findings, we observed increased BACE1 IR on the pial surface in a subset of cases with parenchymal and vascular amyloid pathology (Figure 6). Specifically, increased BACE1 IR around the pia was distinct in sections from 5 cases of the group with prominent intracortical CAA (Figure 6A), whereas BACE1 IR at the same location was minimal in control cases (Figure 6C). Individual BACE1 immunoreactive cells in round or oval shape localized along the pia, apparently lacking processes typical of neuronal or glial cells (inserts in Figure 6A). In adjacent sections, amyloid deposition was present on the cortical surface and also in nearby vascular profiles in these same cases, in contrast to controls (Figure 6B,D).

The finding of concurrent increases of BACE1 and Aβ IR along the pia allowed an opportunity for biochemical verification of the protein change using isolated leptomeningeal samples (Figure 6E,F). Extracts of the leptomeningeal samples over the temporal neocortex of the anatomically verified amyloid and control cases were assayed (Figure 6G-I). BACE1 protein levels were elevated in the lysates of the amyloidosis group (134.2 ± 19.2% of GADPH levels, mean ± SD) relative to control (95.0 ± 11.1%) (P = 0.021, n = 5, Paired *t* test, same test below) (Figure 6G,H). APP β-cleavage activity was increased in the amyloidosis cases (174.9 ± 35.5%) relative to controls (100 ± 16.9%) (P = 0.001) (Figure 6H). Levels of APP C99 were also higher in the amyloidosis group (103.2 ± 32.3% of GADPH levels) than control (70.9 ± 18.3%) (P = 0.009) (Figure 6G,H). Moreover, Aβ42 levels were elevated for about 2 fold in the amyloidosis cases (221.6 ± 86.2 pM/ml) compared to controls (63.2 ± 8.1 pM/ml) (P = 0.013) (Figure 6I).

Immunocytochemistry was used to determine if BACE1 and other Aβ producing proteins could be endogenously expressed in meningeal cells. Meningeal biopsies (from 6 patients) were available for primary cell culture to address this question. In initial assays the cultured cells derived from 3 cases showed 70-90% purity of human meningeal cells, as characterized by a polygonal morphology (Figure JE) and the expression of fibronectin (FN), and a lack of labeling for GFAP (astrocytic marker), CD11 (endothelial marker) or MAP2 (neuronal marker) [44]. We then carried out double immunofluorescence to check the expression of amyloidogenic protein. These experiments revealed BACE1 (Figure 6K,L), APP (Figure 6M) and PS1 (data not shown) labeling in the meninge-derived cells.

## Discussion

### BACE1 elevation in vascular endothelia and perivascular neurites in CAA

Several previous studies have reported that vascular endothelia are capable of producing Aβ in vitro, as characterized with the brain microvascular endothelial cell (BMEC) and the human umbilical vein endothelial cell (HUVEC) lines [27-29]. Notably, vascular endothelial cells express the three alternatively spliced APP mRNA isoforms APP695, APP751 and APP770, whereas neurons express APP695. At the protein level, APP770 is the major variant expressed by the vascular endothelial cells [45]. In the present study, endothelial cells are identified to exhibit increased BACE1 IR in postmortem human brains with cerebral amyloidosis, as compared to controls. Immunocytochemical and biochemical analyses indicate the presence of the amyloidogenic proteins in vascular cells, with BACE1 being detected in endothelial but not smooth muscle cells. Vascular APP migrates at a slightly heavier position than the brain counterpart in immunoblot, consistent with the notion that the former likely represents the APP770 variant [45]. APP β-CTF and APP β-cleavage enzyme activity are detectable in vascular biopsies and primary cells.

We also demonstrate here novel BACE1 immunoreactive neuritic elements in the perivascular region in aged human brains with CAA, which may extend into the paravascular parenchyma as well as into the vascular wall. The neuritic nature of these elements is indicated by their sprouting/swelling configuration and colocalization with synaptophysin. In line with the BACE1 data, sprouting/dystrophic neurites within and outside the vascular wall are visualized by NADPH-d histochemistry, which labels neuronal processes innervating vasculature in the normal mammalian cerebrum [39-41]. These findings demonstrate that neuritic elements present around cerebral vasculature under physiological conditions could develop into amyloidogenic dystrophic neurites with a potential to sprout into the vascular wall under pathological conditions.

The BACE1 labeled neuritic elements appear to develop in the smooth muscle layer after the aforementioned endothelial BACE1 elevation. The correlative morphometric analysis further suggests that the occurrence of these abnormal neurites in the vascular wall is associated with an increasing Aβ deposition and loss of cells (bisbenzimide nuclear profiles) in the vascular wall. In line with the fact that CAA is commonly associated with microbleeding [5-14], we also find iron deposition at sites with BACE1 labeled dystrophic neurites inside and surrounding the vascular wall.

### BACE1 elevation in meningeal cells associated with leptomeningeal amyloidosis

Leptomeningeal amyloidosis occurs often in concurrent with intracortical CAA in the human brain [3-7]. As leptomeninge is enriched of blood vessels, it is reasonable to consider leptomeningeal amyloidosis a form of CAA. However, leptomeningeal Aβ deposition may spread along the cerebral surface continuously rather than invariably be associated with leptomeningeal vasculature or blood vessels entering the cortex. In the present study we observe BACE1 IR in cellular profiles along the pia, in addition to vascular endothelial cells and perivascular dystrophic neurites. Levels of BACE1 protein, activity and the immediate products C99 are elevated in leptomeningeal lysates from brains with pial amyloidosis relative to controls. Using primary cell culture from human subjects, meningeal cells are found to express BACE1 and other amyloidogenic protein. Therefore, meningeal cells could be one of the cellular sources contributing Aβ that might deposit locally in the leptomeninge.

### A hypothetic model for angiopathic and leptomeningeal β-amyloid deposition

Amyloidosis consists of a wide spectrum of conditions with pathological protein deposition in the central and peripheral systems [46]. Aβ deposition has been so far predominantly, if not exclusively, characterized in the central nerve system. Here we show BACE1 expression in vascular endothelia, perivascular dystrophic neurites and meningeal cells at sites of vascular and leptomeningeal amyloidosis in aged human cerebrum. The finding of BACE1 elevation in perivascular neurites and meningeal cells appears of particular interest, and may imply an anatomic basis underlying the brain specificity of vascular and leptomeningeal β-amyloidosis.

**Figure 7:**
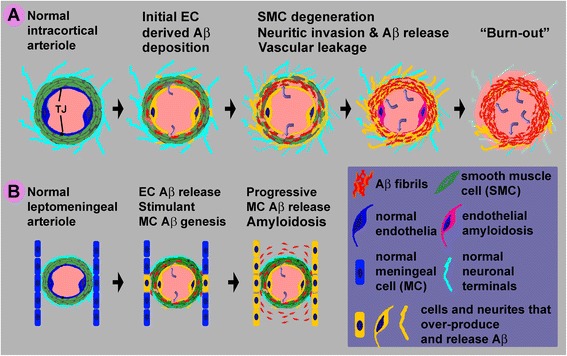
Schematic illustration of a hypothetic model for BACE1 elevation in vascular and brain-specific cellular elements in angiopathic (panel **A**) and leptomeningeal (panel **B**) amyloidosis. Arteriolar profile is used here to construct the model to show the major relevant cellular components and pathological events. BACE1 elevation first occurs in endothelial cells (EC), resulting in Aβ rise and aggregation in the smooth muscle cell (SMC) layer. This is followed by the damage of tight junctions (TJ) and blood brain barrier, causing leakage of blood contents into the SMC layer (curved arrows). The Aβ products and/or blood infiltration then induce SMC degeneration, and further a reactive response of the perivascular axonal terminals, manifested as aberrant sprouting and dystrophy. The invasion of the Aβ producing dystrophic neurites into the vascular wall leads to a vicious cycle of amyloidosis, cell degeneration and microbleeding. This process may end up with a “burnout” stage whereby the ECs, SMCs and dystrophic neurites all degenerate (panel **A**). For leptomeningeal amyloidosis, we propose that the initial events (endothelial Aβ overproduction, Aβ aggregation, BBB breakdown, and SMC degeneration) potentiate BACE1 expression in the nearby meningeal cells. A vicious cycle of pathogenesis in the blood vessel and meninge collectively contribute to the spread of amyloidosis along the leptomeninge (panel **B**).

Although definitively revealing the onset and evolution of a typical pathology would require time-lapsing observation in vivo, staging cellular/histological alterations is a general practice in pathology and clinic medicine. Based on the current findings as discussed above, we propose a dual-origin pathogenic model for angiopathic and leptomeningeal Aβ deposition in the human brain (Figure 7). Stimulated by certain stress factors, the endothelial cells (EC) start to over-express BACE1 (or APP as well), resulting in increased Aβ production (Figure 7A). The EC-derived Aβ peptides are released into blood as well as inside the vascular wall, causing a damage of the tight junctions (TJs) and the blood brain barrier. Vascular leakage and the soluble and insoluble Aβ products cause smooth muscle cell (SMC) degeneration. These events (cellular toxicity and space-emptying effect) trigger a reactive response of the perivascular axonal terminals, manifested by aberrant sprouting and dystrophy, which is inherently associated with BACE1 upregulation [25]. As the dystrophic neurites invade into the vascular wall, they contribute more Aβ products into the local environment. This eventually leads to a vicious cycle of amyloidosis, cell degeneration and microbleeding. At the end stage, cell components including the SMCs, ECs and the dystrophic neurites may all degenerate (“burn-out”), leaving the original vascular site as a niche with concentrated insoluble Aβ products. For the involvement of meningeal cells in leptomeningeal amyloidosis, the initial events may be similar to the above (Figure 7B). Vascular leakage and Aβ products then potentiate BACE1 expression in meningeal cells in the outer and inner barrier layers of the arachnoid and on the pia (not illustrated), resulting in Aβ overproduction and accumulation in the leptomeninge. A vicious pathogenic cycle eventually leads to a spreading of amyloidosis along the brain surface.

## Conclusions

The present study shows increased BACE1 immunoreactivity in vascular endothelia, perivascular dystrophic neurites and meningeal cells in partnership with angiopathic and leptomeningeal Aβ deposition in postmortem human brains. Elevations of BACE1 protein and enzymatic activity in association with leptomeningeal amyloidosis are verified biochemically. Primary cell cultures from human biopsies indicate an endogenous expression of BACE1 and other amyloidogenic proteins in vascular endothelial and meningeal cells. Together, these findings suggest that both vascular and brain-specific cellular components (neuronal terminals and meningeal cells) may contribute to angiopathic and leptomeningeal amyloidosis.
